# Impact of the Laparoscopic Approach on Liver Function Tests: Comparison of Elective Biliary and Non-biliary Procedures

**DOI:** 10.7759/cureus.81500

**Published:** 2025-03-31

**Authors:** Sadia A Baksh, Shah Muhammad, Usra Parvez, Bushra Shirazi, Muhammad A Khan

**Affiliations:** 1 General Surgery, Sindh Institute of Urology and Transplantation, Karachi, PAK; 2 Hepato-Pancreato-Biliary (HPB) and Transplant Surgery, Sindh Institute of Urology and Transplantation, Karachi, PAK; 3 General Surgery, Sindh Institute of Medical Sciences, Karachi, PAK; 4 Organ Recovery Surgery, Network for Hope, Louisville, USA

**Keywords:** intra-abdominal pressure (iap), laparoscopic surgery, liver function tests, pneumoperitoneum, robotic surgery

## Abstract

Introduction: This study aims to explore if laparoscopic surgery impacts liver function tests (LFTs). This study compares LFT changes following elective biliary and non-biliary laparoscopic procedures to determine if the laparoscopic approach itself, rather than underlying biliary pathology, contributes to these alterations.

Methods: This prospective, observational study (July 2023 to June 2024) included 116 American Society of Anesthetists (ASA) grades I and II patients undergoing laparoscopic procedures with normal preoperative LFTs. Exclusion criteria included pre-existing liver disease or conversion to open surgery. The LFTs (aspartate aminotransferase (AST), alanine aminotransferase (ALT), alkaline phosphatase (ALP), gamma-glutamyl transferase (GGT), and bilirubin) were measured preoperatively, at 24 hours, and 72 hours postoperatively. Statistical analysis included Friedman ANOVA, t-tests, and repeated measures ANOVA to compare LFT changes between groups and assess the effect of surgical duration.

Results: A total of 90 patients underwent a biliary procedure, while 26 patients had a non-biliary procedure. Significant postoperative changes in LFTs were observed, with total bilirubin (TB), AST, ALT, ALP, and GGT significantly increasing at 24 hours (p<0.001) before declining at 72 hours. Preoperative TB and GGT were higher in the biliary group (p=0.034 and p=0.023, respectively). The AST was significantly higher at 24 hours for biliary procedures (p <0.001), with a similar level at baseline. Procedure duration showed a significant association with GGT levels at 24 hours only (p=0.031).

Conclusion: Laparoscopic surgery results in transient derangement of LFTs, peaking at 24 hours postoperatively, irrespective of biliary or non-biliary indication. These findings underscore the importance of recognizing this transient effect and suggest routine intervention based solely on these changes may be unwarranted.

## Introduction

Laparoscopic surgery has emerged as a cornerstone of modern minimally invasive surgical practice, offering advantages such as reduced postoperative pain, shorter hospital stays, and faster recovery times compared to traditional open procedures. These benefits are particularly impactful in low- and middle-income countries (LMICs), where resource constraints necessitate efficient and cost-effective healthcare solutions [1]. However, despite its widespread adoption and proven benefits, laparoscopic surgery is not without its challenges. One notable concern is its potential impact on liver function, as evidenced by alterations in liver function tests (LFTs) following these procedures [2].

The physiological changes induced by laparoscopic surgery, particularly the creation of a pneumoperitoneum using carbon dioxide (CO₂) insufflation, likely play an important role in these alterations. Elevated intra-abdominal pressure (IAP) during pneumoperitoneum has been shown to impair hepatic, cardiovascular, respiratory, and renal functions, with the degree of impairment often correlating with the duration and pressure levels maintained during the procedure [3, 4]. Studies have demonstrated that increased IAP can lead to a significant reduction in hepatic blood flow, with a 39% decrease observed when IAP rises from 10 mmHg to 15 mmHg [5]. This reduction in perfusion may contribute to transient hepatic dysfunction, as reflected by elevated serum liver enzyme levels postoperatively [6].

The impact of laparoscopic procedures on LFTs has been studied previously, particularly in the context of cholecystectomy. Research comparing laparoscopic and open cholecystectomy has shown a more pronounced increase in hepatic enzymes (aspartate aminotransferase (AST), alanine aminotransferase (ALT), and alkaline phosphatase (ALP)) in laparoscopic cases, with levels typically peaking on postoperative day (POD)-1 and returning to baseline by POD-7. In contrast, open cholecystectomy is associated with milder changes, with most patients achieving normal LFTs by POD-2 [2]. These findings suggest that the laparoscopic approach itself, rather than biliary pathology alone, may be a significant contributor to postoperative liver enzyme elevation.

Additional factors, such as the use of electrocautery devices, gallbladder traction, and patient positioning during surgery, have also been implicated in these changes. For instance, studies have reported significant rises in ALT and AST levels following the use of both monopolar and harmonic scalpels, even in procedures where the surgical focus is distant from the liver [7]. Furthermore, the combination of general anesthesia, head-up positioning, and potential vascular compression during surgery may exacerbate transient hepatic dysfunction [8, 9]. Given that deranged LFTs may be associated with increased patient morbidity and delayed recovery, understanding the mechanisms and patterns of these changes is important for optimizing postoperative care [10].

This study aims to address three key objectives: (1) demonstrate if the laparoscopic procedures result in changes in the postoperative LFTs, (2) determine if this change is similar for the biliary and non-biliary procedures or otherwise, and (3) evaluate the influence of the procedure duration on these alterations. By investigating these factors, we seek to generate generalizable data that can serve as a reference for clinicians.

## Materials and methods

This cross-sectional, observational study was conducted after the ethical review committee of the Sindh Institute of Urology and Transplantation (SIUT), Karachi, Pakistan, issued approval (approval number: 474). The study was conducted from July 2023 to June 2024. Patients aged between 18 and 65 years, admitted for laparoscopic procedures with normal LFTs and American Society of Anesthetists (ASA) grades I and II status, were offered participation and included if they consented. Patients with deranged liver enzyme levels before an operation, suspected or coexisting chronic liver diseases, converted to open procedure, and with a history of hematological or hepatobiliary disorders were excluded from the study. A total of 116 patients constituted the study population based on the consecutive sampling method.

All patients underwent standardized clinical and laboratory evaluation, including ultrasonography, complete hemogram, and hepatic function tests (AST, ALT, ALP, gamma-glutamyl transferase (GGT), and bilirubin). The normal ranges for liver enzymes were as follows: AST <42 U/L, ALT <41 U/L, ALP 50-136 U/L, GGT <40 U/L, and total bilirubin (TB) <1 mg/dL.

During the surgery, the carbon dioxide (CO^2^) pneumoperitoneum pressure was maintained between 8 mmHg and 14 mmHg. Demographic information, including age, ASA grade, duration of anesthesia, and surgery, was recorded in a predesigned proforma. A standardized institutional anesthetic drug protocol was used for all patients. Liver function tests were performed 24 hours before the surgery, at 24 hours, and at 72 hours after the surgery.

For comparative analysis, patients were categorized into those either undergoing a laparoscopic procedure involving the biliary tree or liver (for instance, cholecystectomy) or a non-biliary procedure (for example, diagnostic laparoscopy). This categorization was done following the collection of data to distinguish potential effects of traction and electrocautery around the liver and biliary tree, leading to a derangement of postoperative LFTs as opposed to the pneumoperitoneum, intra-abdominal pressure, and modification of vascular flows during laparoscopy as drivers of changes in LFTs.

The data were analyzed with IBM SPSS Statistics software version 21.0 (IBM Corp., Armonk, NY) for statistical analysis. Mean ± SD was estimated for quantitative variables like age and pre- and post-operative LFT level. Qualitative variables were expressed in terms of frequency or percentage. Comparison of pre- and postoperative LFTS was based on the related-samples Friedman two-way ANOVA by the Ranks method. The laboratory outcomes for biliary and non-biliary procedures were compared using an independent sample t-test with equal variances not assumed. Repeated measure ANOVA with multiple testing of the Bonferroni test was applied to assess the change in LFT levels when comparing for stratified duration of surgery (i.e., < one hour, between one to two hours, and > two hours).

## Results

Among the 116 patients included in the study, there were 35 male and 81 female patients. The average age of the patients was 43.66±11.95 years. Almost 83% of the patients were ASA grade II. Regarding comorbidities, 52 of 116 (44.83%) were hypertensive, 12 (10.4%) were diabetic, and 64 (55.17%) had no known comorbidities. Among all the patients, 90 (77.6%) had cholecystectomy, 12 (10.3%) had diagnostic laparoscopy or mesenteric lymph nodal biopsy, four (3.4%) patients had staging laparoscopy as a part of various oncological procedures, two (1.7%) had appendectomy, four (3.4%) had transabdominal preperitoneal (TAPP) repair for inguinal hernias, and four (3.4%) had laparoscopic low-anterior resection. The following results are summarized based on the stated objectives.

Changes in LFTs post laparoscopic procedures

A significant change in LFTs was observed following laparoscopic procedures. Total bilirubin nearly doubled at 24 hours postoperatively (p<0.001), before decreasing to nearly baseline levels at 72 hours. A similar trend was noted for AST and ALT, which significantly increased at 24 hours, with AST showing a 25% and ALT more than 30% higher level than baseline (p<0.001 for both), followed by a decline at 72 hours. Both ALP and GGT also exhibited significant postoperative changes, peaking at 24 hours before returning closer to baseline at 72 hours (p<0.001). Direct bilirubin (DB) showed no significant variation across time points (p=0.67) (Table 1). 

**Table 1:** Comparison of pre- and postoperative mean liver function test (LFT) levels at standardized time intervals (n=116) ^1 ^Based on related-samples Friedman two-way ANOVA by ranks; *pre- vs. 24 hours postoperative; p<0.01; ^†^24 hours postoperatively vs. 72 hours postoperatively; p<0.01; pre vs. 72 hours postoperatively>0.05; no significant difference was observed between 72 hours and preoperative LFT levels.

LFTs	Normal values unit	24 hours preoperative	24 hours postoperative	72 hours postoperative	p-value^1^
Total bilirubin (TB)	<1 mg/dL	0.44±0.25	0.82±0.42*	0.50±0.24†	<0.001
Direct bilirubin (DB)	<1 mg/dL	0.16±0.09	0.22±0.21*	0.16±0.08†	0.67
Aspartate aminotransferase (AST)	<42 U/L	29.89±15.58	41.92±21.67*	31.87±14.38†	<0.001
Alanine aminotransferase (ALT)	<41 U/L	31.56±17.77	45.59±20.61*	32.40±16.43†	<0.001
Alkaline phosphatase (ALP)	50-136 U/L	102.37±33.77	113.01±41.35*	94.81±29.33†	<0.001
Gamma-glutamyl transferase (GGT)	<40 U/L	36.45±24.75	49.20±36.91*	41.34±21.85†	<0.001

Comparison of biliary vs. non-biliary procedures

A subgroup analysis comparing biliary and non-biliary laparoscopic procedures revealed some notable differences. Preoperative TB levels were significantly higher in the biliary group (0.45±0.26 mg/dL) compared to the non-biliary group (0.36±0.16 mg/dL, p=0.034). However, at 24 and 72 hours postoperatively, the differences between the two groups were not statistically significant (p=0.179 and p=0.166, respectively). Similarly, baseline GGT was higher in the biliary group (p=0.023) but not after 24 or 72 hours of surgery. Among postoperative tests, ALP was similar for both groups at baseline, trended up at 24 hours similarly in both groups, but showed a significantly less decline in the biliary compared to the non-biliary group at 72 hours (p=0.004). For AST, there was a significantly higher rise for biliary procedures at 24 hours (p <0.001), with a similar level at baseline. The AST level also reached similar levels at 72 hours between the two groups. Other LFT parameters did not exhibit significant differences between the two surgical categories (Table 2).

**Table 2:** Comparison of LFT results at baseline, 24 and 72 hours hours postoperatively between biliary and non-biliary procedures ^1 ^Based on independent sample T-test with equal variances not assumed. LFT: liver function tests; TB: total bilirubin; DB: direct bilirubin; ALP: alkaline phosphatase; ALT: alanine transaminase; AST: aspartate transaminase; GGT: gamma-glutamyl transferase

LFTs	Normal values unit	Surgery type		T values	p-value^1^
		Biliary surgery	Non-biliary surgery		
		Mean +s.d.	Mean +s.d.		
TB	<1 mg/dL	0.45 +0.26	0.36 +0.16	2.160	0.034
TB-24 hours postoperatively	<1 mg/dL	0.83 +0.42	0.69 +0.47	1.369	0.179
TB-72 hours postoperatively	<1 mg/dL	0.49 +0.23	0.58 +0.30	-1.414	0.166
DB	<1 mg/dL	0.16 +0.09	0.17 +0.15	-0.324	0.749
DB-24 hours postoperatively	<1 mg/dL	0.22 +0.20	0.27 +0.33	-0.735	0.468
DB-72 hours postoperatively	<1 mg/dL	0.15 +0.09	0.17 +0.14	-0.688	0.496
ALP	50-136 U/L	102 +35	102 +23	0.000	1.000
ALP-24 hours postoperatively	50-136 U/L	114 +42	101 +32	1.693	0.096
ALP-72 hours postoperatively	50-136 U/L	97 +29	80 +24	3.029	0.004
ALT	<41 U/L	32 +18	27 +10	1.832	0.071
ALT-24 hours postoperatively	<41 U/L	46 +21	42 +19	0.923	0.361
ALT-72 hours postoperatively	<41 U/L	33 +17	28 +15	1.452	0.154
AST	<42 U/L	30 +15	30 +17	0.000	1.000
AST-24 hours postoperatively	<42 U/L	43 +22	36 +16	2.794	<0 .001
AST-72 hours postoperatively	<42 U/L	32 +15	29 +13	1.000	0.323
GGT	<40 U/L	37 +26	28 +14	2.320	0.023
GGT-24 hours postoperatively	<40 U/L	51 +37	35 +36	1.984	0.054
GGT-72 hours postoperatively	<40 U/L	42 +22	33 +22	1.837	0.073

Effect of procedure duration on LFT changes

**Figure 1:**
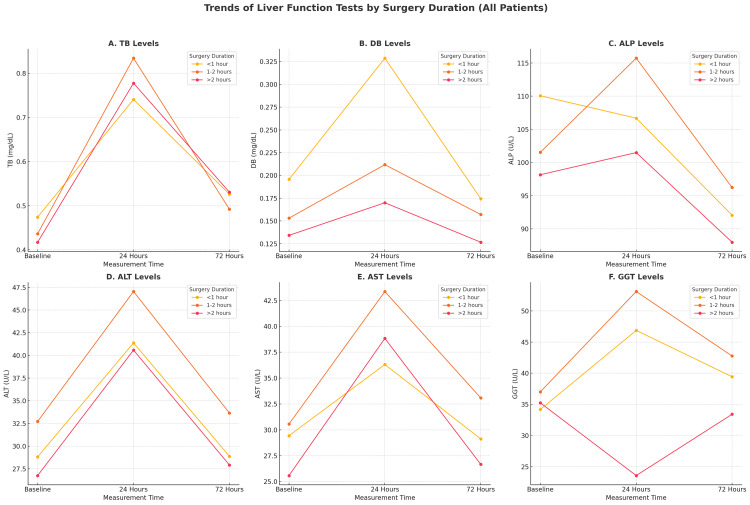
Mean LFT plots for duration of surgery (<1, 1-2 and >2hours) Trends of liver function test (LFT) results by stratified surgery duration (all patients) are as follows: Panel A: Total bilirubin (TB): Mean serum TB levels (mg/dL) across measurement times (baseline, 24 hours, and 72 hours) stratified by surgical duration groups (< one hour, one to two hours, > two hours); Panel B: Direct bilirubin (DB): Mean serum DB levels (mg/dL) at baseline, 24 hours, and 72 hours, illustrating changes according to the duration of surgery; Panel C: Alkaline phosphatase (ALP): Mean serum ALP levels (U/L) measured at three different time intervals post-surgery, grouped by surgical duration categories; Panel D: Alanine transaminase (ALT): Mean serum ALT levels (U/L) evaluated at baseline, 24 hours, and 72 hours, showing trends by surgery duration groups; Panel E: Aspartate transaminase (AST): Mean serum AST levels (U/L) assessed at the three time points, highlighting variations related to different surgical durations.

The impact of surgical duration on postoperative LFT changes was analyzed using ANOVA. No significant association was observed between surgery duration and changes in TB, DB, AST, ALT, or ALP (p>0.05 for all). However, GGT levels at 24 hours demonstrated a significant difference based on surgical duration (p=0.031), suggesting a potential transient effect of longer procedures on this enzyme (Table 3). A graphic summary of changes in each parameter of LFTs in relation to the stratified duration of surgery is presented in Figure 1. In general, it demonstrates that the correlation of the duration of surgery with each of the biochemical variables is not intuitive and varies for each of these in distinct patterns.

**Table 3:** Comparison of LFTs for stratified duration of surgery based on ANOVA This comparison is based on an analysis of variance between three stratified operative duration groups, viz., <one hour, between one to two hours, and >two hours. LFT: liver function tests; TB: total bilirubin; DB: direct bilirubin; ALP: alkaline phosphatase; ALT: alanine transaminase; AST: aspartate transaminase; GGT: gamma-glutamyl transferase

LFTs		Mean square	F	p-value
TB	Between groups	.013	0.204	.815
	Within groups	.063		
TB-24 hours postoperatively	Between groups	.069	0.381	.684
	Within groups	.181		
TB-72 hours postoperatively	Between groups	.014	0.234	.792
	Within groups	.059		
DB	Between groups	.016	1.592	.208
	Within groups	.010		
DB-24 hours postoperatively	Between groups	.112	2.535	.084
	Within groups	.044		
DB-72 hours postoperatively	Between groups	.008	0.927	.399
	Within groups	.009		
ALP	Between groups	609.319	0.530	.590
	Within groups	1150.446		
ALP-24 hours postoperatively	Between groups	1439.550	0.839	.435
	Within groups	1715.326		
ALP-72 hours postoperatively	Between groups	428.451	0.494	.612
	Within groups	867.442		
ALT	Between groups	257.996	0.814	.446
	Within groups	317.032		
ALT-24 hours postoperatively	Between groups	385.736	0.906	.407
	Within groups	425.774		
ALT-72 hours postoperatively	Between groups	288.506	1.070	.346
	Within groups	269.617		
AST	Between groups	132.487	0.541	.584
	Within groups	244.802		
AST-24 hours postoperatively	Between groups	400.417	0.850	.430
	Within groups	471.163		
AST72	Between groups	287.100	1.397	.252
	Within groups	205.494		
GGT	Between groups	64.024	0.103	.902
	Within groups	622.590		
GGT-24 hours postoperatively	Between groups	4654.455	3.567	.031
	Within groups	1304.775		
GGT-72 hours postoperatively	Between groups	494.523	1.036	.358
	Within groups	477.370		

## Discussion

This study investigated the impact of laparoscopic procedures on LFTs, comparing biliary and non-biliary procedures and evaluating the influence of surgical duration. Our key finding is the demonstration of transient LFT derangement following laparoscopic surgery, characterized by a significant increase in TB, AST, ALT, ALP, and GGT at 24 hours postoperatively, followed by a return towards baseline levels by 72 hours. Direct bilirubin did not exhibit significant changes. This transient elevation of liver enzymes, predominantly observed within the first 24 hours, aligns with previous research [11-18]. These prior studies, largely focusing on laparoscopic cholecystectomy, have consistently reported similar trends of postoperative LFT elevation. Adding information regarding the changes in the LFTS following non-biliary surgical procedures, our study supports the notion that the laparoscopic approach itself, rather than biliary pathology alone, contributes to these changes.

Our comparison of biliary and non-biliary procedures revealed a statistically significant difference in preoperative TB and GGT levels, with the biliary group exhibiting higher values. However, postoperative TB and GGT levels, as well as most other LFT parameters, were comparable between the two groups. This suggests that while preoperative biliary status might influence baseline TB, the postoperative LFT changes are likely driven by factors common to both biliary and non-biliary laparoscopic procedures, such as pneumoperitoneum and surgical manipulation.

A higher rise in ALT at 24 hours for biliary procedures, with a return to similar levels at 72 hours, suggests a transient difference likely owing to higher direct energy transfer to the liver as compared to non-biliary procedures. Similarly, a slower decline in ALP at 72 hours also suggests possible decreased biliary transit owing to a similar effect.

The impact of surgical duration on LFTs was also investigated. While most LFT parameters were not significantly affected by procedure duration, GGT levels at 24 hours showed a significant association, suggesting a potential transient effect of longer procedures on this specific enzyme. Sharma et al. [18] also found that longer duration of surgery was associated with more alterations in mean serum bilirubin in laparoscopic cholecystectomy patients. This finding warrants further investigation with larger sample sizes and more granular categorization of surgical durations.

This study has some limitations. The sample size, while adequate for detecting overall trends, may have limited the power to detect subtle differences between subgroups or the impact of very short or very long procedures. Furthermore, the study was conducted in a single center, which might limit the generalizability of the findings. Future research with larger, multicenter cohorts and more detailed assessment of intraoperative parameters, such as IAP levels and specific surgical maneuvers, would provide a more comprehensive understanding of the mechanisms involved. Additionally, evaluating the correlation between LFT changes and clinical outcomes, including postoperative recovery and complications, would be valuable. Investigating the potential benefits of minimizing pneumoperitoneum pressure or optimizing surgical techniques to mitigate LFT derangement also represents an important avenue for future research [19-21].

## Conclusions

In conclusion, our study demonstrates that laparoscopic surgery, irrespective of being biliary or non-biliary in nature, is associated with a transient derangement of liver function tests, typically peaking at 24 hours postoperatively and returning towards baseline by 72 hours. While preoperative biliary status influences baseline total bilirubin and gamma-glutamyl transferase, the postoperative LFT changes are primarily driven by factors inherent to the laparoscopic approach. Procedure duration appears to have minimal impact on most LFT parameters, apart from a potential transient effect on GGT. These findings underscore the importance of recognizing this transient LFT derangement in the postoperative period following laparoscopic surgery and suggest that routine intervention based solely on these changes may be unwarranted. However, further research is needed to clarify the impact of surgical duration, standardize intraoperative factors, and evaluate the clinical significance of these biochemical alterations on patient outcomes.
